# MerTK inhibition by RXDX-106 in MerTK activated gastric cancer cell lines

**DOI:** 10.18632/oncotarget.22394

**Published:** 2017-11-11

**Authors:** Jung Eun Kim, Youjin Kim, Gary Li, Seung Tae Kim, Kyung Kim, Se Hoon Park, Joon Oh Park, Young Suk Park, Ho Yeong Lim, Hyuk Lee, Tae Sung Sohn, Kyoung-Mee Kim, Won Ki Kang, Jeeyun Lee

**Affiliations:** ^1^ Division of Hematology-Oncology, Department of Medicine, Samsung Medical Center, Sungkyunkwan University School of Medicine, Seoul, Korea; ^2^ Ignyta, Inc., San Diego, CA, USA; ^3^ Department of Pathology and Translational Genomics, Samsung Medical Center, Sungkyunkwan University School of Medicine, Seoul, Korea

**Keywords:** MerTK, gastric cancer, patient-derived tumor cells

## Abstract

RXDX-106 is a potent and selective type II pseudo-irreversible (slow off-rate) inhibitor of TYRO3, AXL, MER and c-MET. MER tyrosine kinase (MerTK) is expressed in a variety of malignancies, including gastric cancer (GC). The oncogenic potential of MerTK is supported by various lines of evidence. First, we surveyed 10 GC cell lines for MerTK protein overexpression and MerTk phosphorylation. We next evaluated the change of downstream signaling molecules including (p)-ERK and (p)-AKT, following RXDX-106 treatment. We also investigated the effect of RXDX-106 in patient-derived cell lines to mimic the *in vivo* condition. The prevalence of MerTK protein overexpression was evaluated in 229 cancer tissue specimens. We have found that MerTK inhibitor treatment resulted in considerable inhibition of cell growth and downstream signaling. In addition, MerTK phosphorylation, not total MerTK expression, is likely more predictive of therapeutic success. p-MerTK protein overexpression by IHC was found in 18% (17/87) of GC patients. Lastly, RXDX-106 inhibited cell proliferation in MerTK activated gastric cancer cell line. These findings provide further evidence of oncogenic roles for MerTK in GC, and demonstrate the importance of kinase activity for MerTK tumorigeneicity and validate RXDX-106, a novel MerTK inhibitor, as a potential therapeutic agent for treatment of GC.

## INTRODUCTION

Gastric cancer (GC) is a heterogeneous disease and recent effort has been focused on exploring subsets of patients who may potentially benefit from molecularly targeted agents [1]. Despite of tremendous effort in attempting to prove the survival benefit from targeted agents in a “target” population (i.e. MET inhibitor in MET overexpressed gastric cancer patients), many trials have failed to show survival benefit [2]. The survival outcome for metastatic gastric cancer patients is still near one year after diagnosis and thus, exploration of novel targets in “target” population in GC is urgently needed [2].

MER proto-oncogene tyrosine kinase (MerTK) belongs to the family of TYRO3, AXL, MER (collectively, TAM) receptor tyrosine kinases (RTKs) which are normally expressed in macrophages, dendritic cells and natural killer cells [3]. MerTK overexpression has been demonstrated in several cancer types such as melanoma [4, 5], lung cancer [6], prostate cancer, glioblastoma [7, 8], HCC [9], and head and neck cancer [10]. We recently showed that 8.3% (16 of 192) of GC patients demonstrated strong positive MerTK total protein expression and overall they have poor survival outcome [11]. In addition, we showed that MerTK-overexpressing GC cells were profoundly inhibited after MerTK knockdown, suggesting that MerTK can be a potential novel therapeutic target in GC [11].

It has been shown that one of the first reported MerTK inhibitors, UNC1062, inhibited MerTK phosphorylation and cell proliferation in different tumor cell lines [5]. Recently, RXDX-106 has been developed by Ignyta. RXDX-106 is a potent and selective type II pseudo-irreversible (slow off-rate) inhibitor of TYRO3, AXL, MER and c-MET [12]. Recently, RXDX-106 monotherapy was shown to release the molecular brakes on immune activation in macrophages, NK cells, and T cells, resulting in the repoloraziation of the immune response to elicit an anti-tumor effect [12]. Emerging *in vivo* combination data suggest that RXDX-106 potentiates the activity of anti-PD-1 and anti-CTLA-4 agents.

In this study, we tested the anti-tumor effect of RXDX-106 on MerTK activated gastric cancer cell lines.

## RESULTS

### RXDX-106 inhibits cell proliferation in MerTK activated gastric cancer cell line

**Figure 1:**
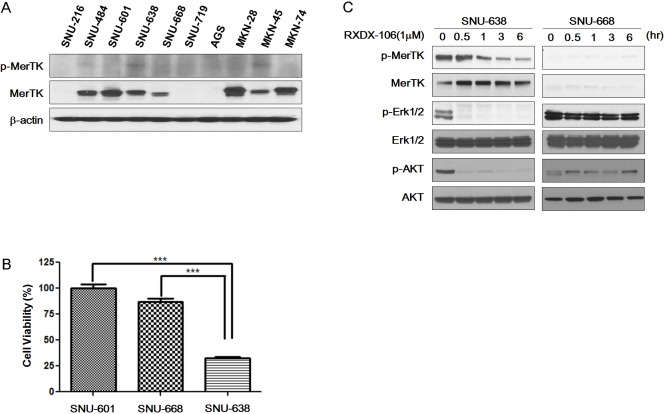
The effect of RXDX-106 in gastric cancer cell lines **(A)** Basal protein expression level of p-MerTK and MerTK, the target of RXDX-106, in gastric cancer cell lines. **(B)** Results of cell viability under 1μM RXDX-106 treatment. Statistical significance was calculated using paired t-test and is indicated with ^***^ for *p*-value < 0.0001. **(C, D)** Molecular change of proliferative signaling molecules and RXDX-106 targeted molecules. For Western blotting, cells were exposed to 1μM RXDX-106 for 3 days or indicated time.

First, we surveyed 10 GC cell lines for MerTK protein overexpression and MerTk phosphorylation (Figure 1A). Of 10 cell lines, MerTK was overexpressed in 7 cell lines (SNU-484, SNU-601, SNU-638, SNU-668, MKN28, MKN45, and MKN74). Of these 7 cell lines, two cell lines (SNU-638 and MKN-45) had elevated levels of phosphor-MerTK, indicating that these cell lines had activated MerTK. Based on the western blotting, we selected SNU-601, 638, 668 and MKN-45 to test the efficacy of RXDX-106. The growth inhibitory effect of RXDX-106 was assessed by the Cell Titer Glo method. RXDX-106 significantly inhibited the growth of MerTK activated SNU-638 cells when compared with SNU-601 and SNU-668 (Figure 1B) (*p*-value < 0.0001).

We next evaluated the change of downstream signaling molecules following 1μM RXDX-106 treatment for 3 days (Figure 1C). To examine the target inhibitory effect of RXDX-106, MerTK and Axl expression was evaluated. In RXDX-106 sensitive cell line SNU-638, both p-MerTK and p-Axl were decreased in RXDX-106 treated cells. In addition, RXDX-106 treatment induced the p-ERK and p-AKT reductions in SNU-638 cells. On the other hand, p-ERK and p-AKT levels were not significantly altered in SNU-668 following RXDX-106 treatment. In the early time point, p-ERK, p-AKT and p-MerTK expression were decreased in SNU-638 following RXDX-106 treatment over time (Figure 1D). However, there's not significantly changed the expression of p-Erk and p-AKT in SNU-668, non-activated MerTK cell line. Collectively, these results suggested that RXDX-106, a MerTK inhibitor, has an anti-proliferative effect by reducing the p-ERK and p-AKT in MerTK activated cancer cells.

### RXDX-106 inhibits MerTK activated GC PDCs

**Figure 2:**
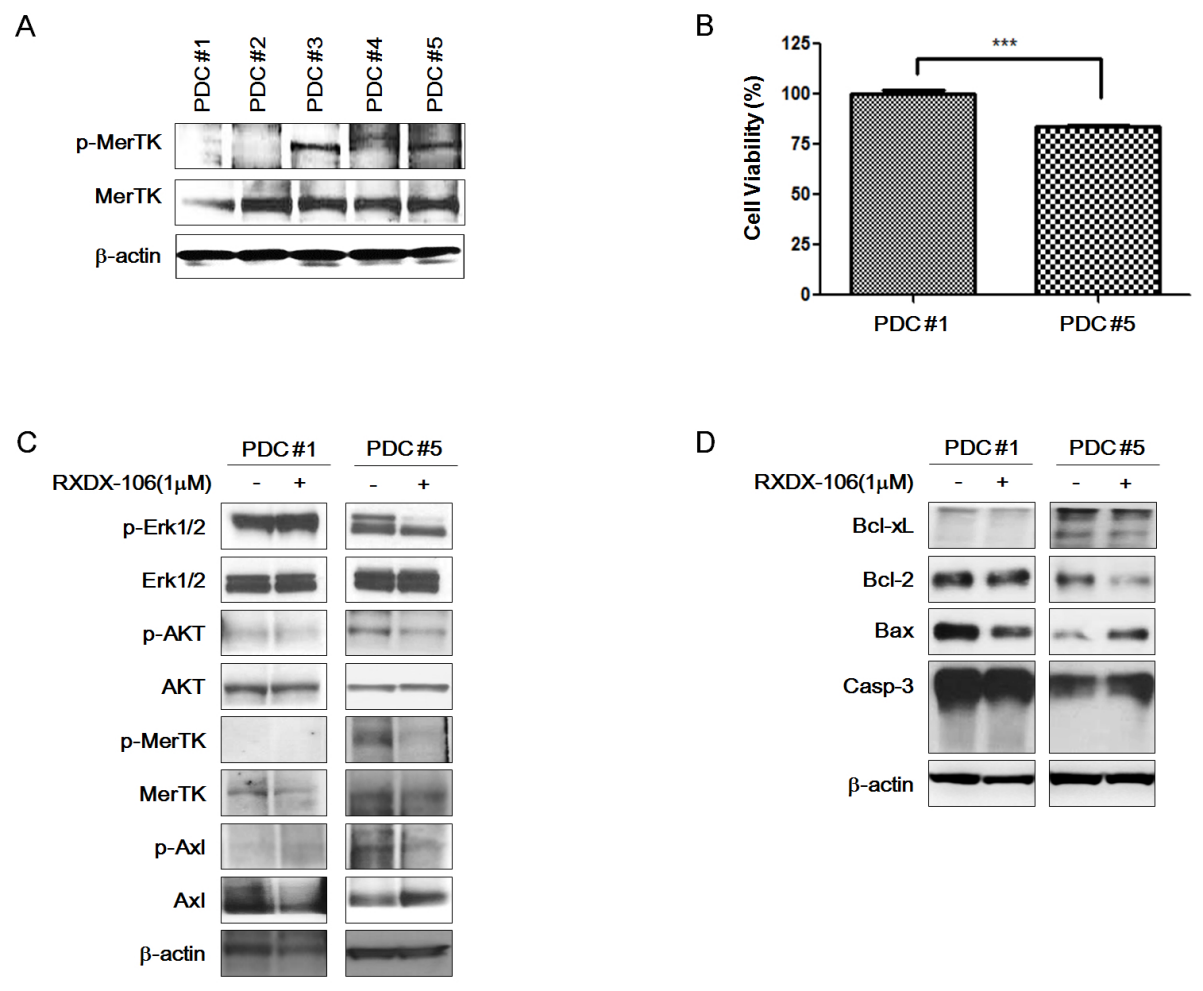
Anti-cancer effect of RXDX-106 in PDCs **(A)** Diversity of p-MerTK and MerTK expression level in PDCs using Western blot analysis. **(B)** Results of cell viability under 1 μM RXDX-106 treatment. Statistical significance was calculated using paired *t*-test and is indicated with ^***^ for *p*-value < 0.0001. **(C)** Molecular change of proliferative signaling molecules and RXDX-106 target molecules in PDCs. **(D)** The change of cell death related proteins in PDCs. For Western blotting, PDCs were exposed to RXDX-106 at 1 μM concentration for 3 days. β-actin used as loading control.

To mimic the *in vivo* conditions, we investigated the effect of RXDX-106 in PDCs. First, we examined the expression level of MerTK and p-MerTK in 5 gastric PDCs (Figure 2A). Total MerTK expression did not significantly differ among the PDCs. However, p-MerTK was detected only in PDC #3 and PDC #5. We selected PDC #1 and PDC #5 as representative PDCs for p-MerTK negative and p-MerTK positive PDC, respectively, for further experiments.

**Figure 3:**
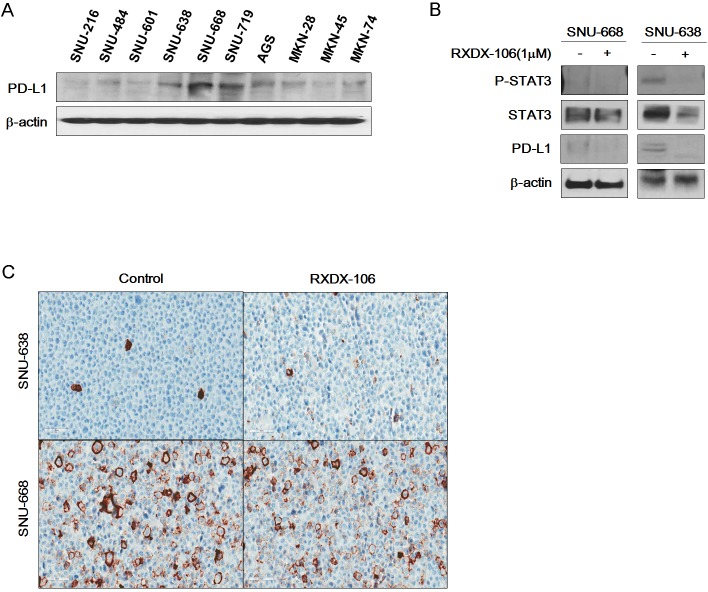
Reduction of PD-L1 expression under RXDX-106 exposure in sensitive cell line **(A)** Basal expression of PD-L1 in gastric cancer cell lines. **(B)** RXDX-106 affects STAT3 and PD-L1. For Western blotting, cells were exposed to RXDX-106 at 1 μM concentration for 3 days. β-actin used as loading control. **(C)** Representative photomicrographs (X40) of SNU-638 and SNU-668 cell pellets stained by the PD-L1 ICC assay.

To examine the effect of RXDX-106, we performed a cell viability test (Figure 2B). At 1μM of RXDX-106, cell viability of PDC #5 is lower than PDC #1 and the difference is statistically significant (*p*-value <0.0001). Similar to the cell line data, p-Erk and p-AKT expression levels were decreased according to RXDX-106 treatment in p-MerTK- expressed PDC #5. P-MerTK and p-Axl, the targets of RXDX-106, were decreased in treated PDC compare to the non-treated one (Figure 2C). In addition, Bcl-xL and Bcl-2 as well known anti-apoptotic molecules, were decreased and Bax, apoptotic signaling molecule was increased in RXDX-106 treated PDC #5. On the other hands, Bcl-xL, Bcl-2 and Bax were not changed according to RXDX-106 treatment in PDC #1 (Figure 2D). Collectively, RXDX-106 shows an anti-proliferative effect through inhibiting proliferative signals and activating apoptotic singals in MerTK activated gastric cancer PDCs. Furthermore, we investigated whether RXDX-106 effects on PD-L1 modulation using Western blot and IHC analysis (Figure 3). Of note, PD-L1 and STAT3 were decreased upon RXDX-106 exposure in MerTK activated cell line (SNU-638) which implicate that the drug may also modulate inflammatory pathway.

### Analysis of p-MerTK expression in various cancer tissue specimens

We aimed to identify the prevalence of MerTK and p-MerTK surface expression among various cancer populations. In total, we quantified MerTK protein expression in tissue samples of 229 patients by immunohistochemistry. We found that 19.5% (17/87) of GC patients had p-MerTK expression. In other cancer types, p-MerTK was found in 18.7% biliary tract cancer (3/16), 27.6% (8/29) of sarcoma, 4.3% (2/46) of genitourinary cancer (Table 1).

**Table 1:** Analysis of phosphor-MerTK expression in various cancer types

Tumor type	p-MERTK		Total N
	Negative	Positive	(n=229)
**Gastric cancer (GC)**	68(78.2%)	17(19.5%)	87
**Genitourinary cancer (GU)**	44(95.7%)	2(4.3%)	46
**Biliary tact cancer (BTC)**	13(81.3%)	3(18.7%)	16
**Pancreatic cancer**	5(100%)	0(0.0%)	5
**HCC**	13(86.7%)	2(13.3%)	15
**Melanoma**	6(66.7%)	3(33.3%)	9
**Sarcoma**	21(72.4%)	8(27.6%)	29
**GIST**	4(66.7%)	2(33.3%)	6
**Small bowel tumor**	4(100%)	0(0.0%)	4
**Others**	11(91.6%)	1(8.3%)	12

## DISCUSSION

In the current study, we have found that 1) MerTK inhibitor treatment resulted in considerable inhibition of cell growth and downstream signaling; 2) MerTK phosphorylation, not total MerTK expression, is likely more predictive of therapeutic success; 3) p-MerTK protein overexpression by IHC was found in 18% (17/87) of GC patients; 4) RXDX-106 inhibited cell proliferation in MerTK activated gastric cancer cell line.

In recent years, therapeutic agents targeting specific molecular aberrations in cancer cells have been effective at prolonging survival in multiple cancer types; however, for the majority of patients with cancer, the oncogenic drivers are complex and identification of additional therapeutic targets has become a major research focus [13, 14].

One potential target is MerTK, a member of the TAM-family of receptor tyrosine kinases, which also includes Axl and Tyro3 [14, 15]. With a defined spectrum of normal expression, MerTK triggers macrophage engulfment of apoptotic material and promotes an anti-inflammatory response by down-modulating pro-inflammatory signals [16]. MerTK functions require the intact tyrosine kinase domain and downstream tyrosine phosphorylation signaling [16]. MerTK is overexpressed or ectopically expressed in various hematological and solid tumors [6–10]. It has since been shown to activate a wide variety of pro-oncogenic signaling pathways in an ever-expanding list of human cancer types. Signaling pathways, including those involving MAPK and p38, PI3K, Janus-activated kinase (JAK)/STAT, FAK/RhoA/MLC2, and Bcl-2 family members, contribute to increased proliferation and migration, and decreased apoptosis and chemosensitivity [14]. Therefore, inhibition of MerTK may provide dual therapeutic effects against MerTK-expressing tumors by reducing cancer cell survival, invasion, and metastasis as well as stimulating antitumor immune responses. Target validation studies, suggest that MerTK inhibition is a viable strategy for decreasing tumor burden in preclinical models. Many studies have used shRNA to show critical oncogenic roles for MerTK in a variety of tumor types [17]. Regarding GC, we recently showed that 8.3% (16 of 192) of GC patients demonstrate strong positive protein expression and they have poor outcome survival [11]. TAM receptors have been previously shown to activate Akt and Erk signaling pathway, thus promoting survival and proliferation of cancer cells [18]. Moreover, on infiltrating monocyte-derived cells including macrophages, immature dendritic cells and natural killer cells, TAMs are known to suppress host tumor immunity and block the expression of tumor-derived antigens which lead to tumor progression [19]. RXDX-106 is a potent inhibitor of TAM activation and function in bone marrow derived macrophages, inhibiting both TAM receptor phosphorylation and TAM-dependent phagocytosis. In this study, we demonstrated that RXDX-106 effectively inhibited cell proliferation of p-MerTK overexpressed GC cells. In addition, our *in vitro* data show that RXDX-106 treatment decreased the number of viable cells in p-MerTK expressed PDC. p-MerTK levels are more significant other than total MerTK expression to predict therapeutic success since these tyrosine kinase proteins undergo auto-phosphorylation for the regulation of their activity. MerTK inhibitor also downregulated endogenous PD-L1 level in few GC cell lines with high endogenous PD-L1 protein level which aligns with previous reports [19]. Thus, future studies should evaluate the phosphorylation status of MerTK in GC tumor tissues to investigate whether MerTK activation frequently occurs and whether it has prognostic or predictive value. Another interesting question is whether combination with anti-PD-L1 antibody would confer additional benefit to MerTK inhibition in GC, given that nivolumab, an anti-PD-L1 antibody has proven efficacy in metastatic GC [20].

Taken together, the findings described here provide further evidence of oncogenic roles for MerTK in GC, demonstrate the importance of kinase activity for MerTK tumorigenicity and validate RXDX-106, a novel MerTK inhibitor, as a potential therapeutic agent for treatment of GC. Future studies are warranted to explore whether MerTK expression and/or activation status in tumor tissues would be correlated with the response of GC patients.

## MATERIALS AND METHODS

### Cell lines

Human gastric cancer cells (MKN-28, MKN-45 and MKN-74) were purchased from the American Type Culture Collection (ATCC, Manassas, VA, USA). Other human gastric cancer cells (SNU-216, SNU-484, SNU-601, SNU-638, SNU-668, SNU-719 and AGS) were purchased from Korean Cell Line Bank (Seoul, South Korea). The medium was changed every 3 days, and all cell lines were maintained at 37°C in a 5% CO_2_-humidifed atmosphere. All the cell lines were banked and passaged for less than 3 months before they were used for experiments.

### Patient-derived cancer cell lines

Primary biopsy or surgical samples were washed with DPBS and cut into small pieces and then transferred into the RPMI1640 containing 120 μg/ml of collagenases, 500μg/ml of dispase (Gibco BRL, Paisley, UK) and 2mg/ml of DNase (Roche, Basel, Switzerland). Small pieces of primary tissue were incubated for 0.5 to 1 hour until dispersed as previously described [21–23]. For gathering the single cells, the samples were centrifuged and the supernatant removed. The collected cells were seeded on plate with RPMI1640 containing 10% FBS, 1% antibiotic-anti-mycotic solution, 0.5 μg/ml of hydrocortisone (Sigma Aldrich), 5 μg/ml of insulin (PeproTech, Rocky Hill, NJ, USA) and 5 ng of EGF (PeproTech, Rocky Hill, NJ, USA). Established patient-derived cell lines were grown primary cell culture media containing the supplements. The medium was changed every 3 days, and all cell lines were maintained at 37°C in a 5% CO_2_-humidifed atmosphere.

### Growth inhibition assay

3000 cells were seeded in 96-well plates and incubated overnight at 37°C in 5% CO_2_. The cells were exposed to increasing concentrations of RXDX-106 for 3 days. Cell proliferation was determined using Cell Titer Glo (Promega, Madison, WI, USA) according to the manufacturer's protocol. The detected luminescent signals were calculated to percentage versus control and IC_50_ value was the defined as the drug concentration needed to inhibit 50% of the cell growth compared to growth of the untreated control cells.

### Tissue microarray construction and IHC stains

Tissue microarrays were constructed using a Beecher Manual Tissue Microarrayer (MTA-1, Beecher Instruments Inc., Wisconsin, USA). All available H&E-stained slides were reviewed, and 4 representative tumor regions were taken from donor formalin-fixed paraffin-embedded blocks using a 0.6-mm core punch, and arrayed into recipient blocks. IHC studies were performed with 4-μm-thick tissue microarray sectionsby using rabbit anti-MerTK (phospho Y681 + Y749) antibody (ab192649, Abcam, Cambridge, USA) and cell line sections by Rabbit Anti-Human PD-L1/CD274 Monoclonal Antibody (M4424, Spring bioscience, Pleasanton, CA, USA). The sections were deparaffinized 3 times in xylene for a total of 15 min and subsequently rehydrated. Immunostaining was performed using a Bond-max autoimmunostainer (Leica Biosystems, Melbourne, Australia) with Bond^TM^ Polymer refine detection, DS9800 (Vision Biosystems, Melbourne, Australia). Briefly, antigen retrieval was achieved by heating samples to 97°C for 20 min in Bond-max ER1 buffer in 97°C for 20 min, blocking endogenous peroxidase activity with 3% hydrogen peroxidase for 5 min, and incubating samples with a 1:200 dilution of primary antibody for 15 min.

**Figure 4:**
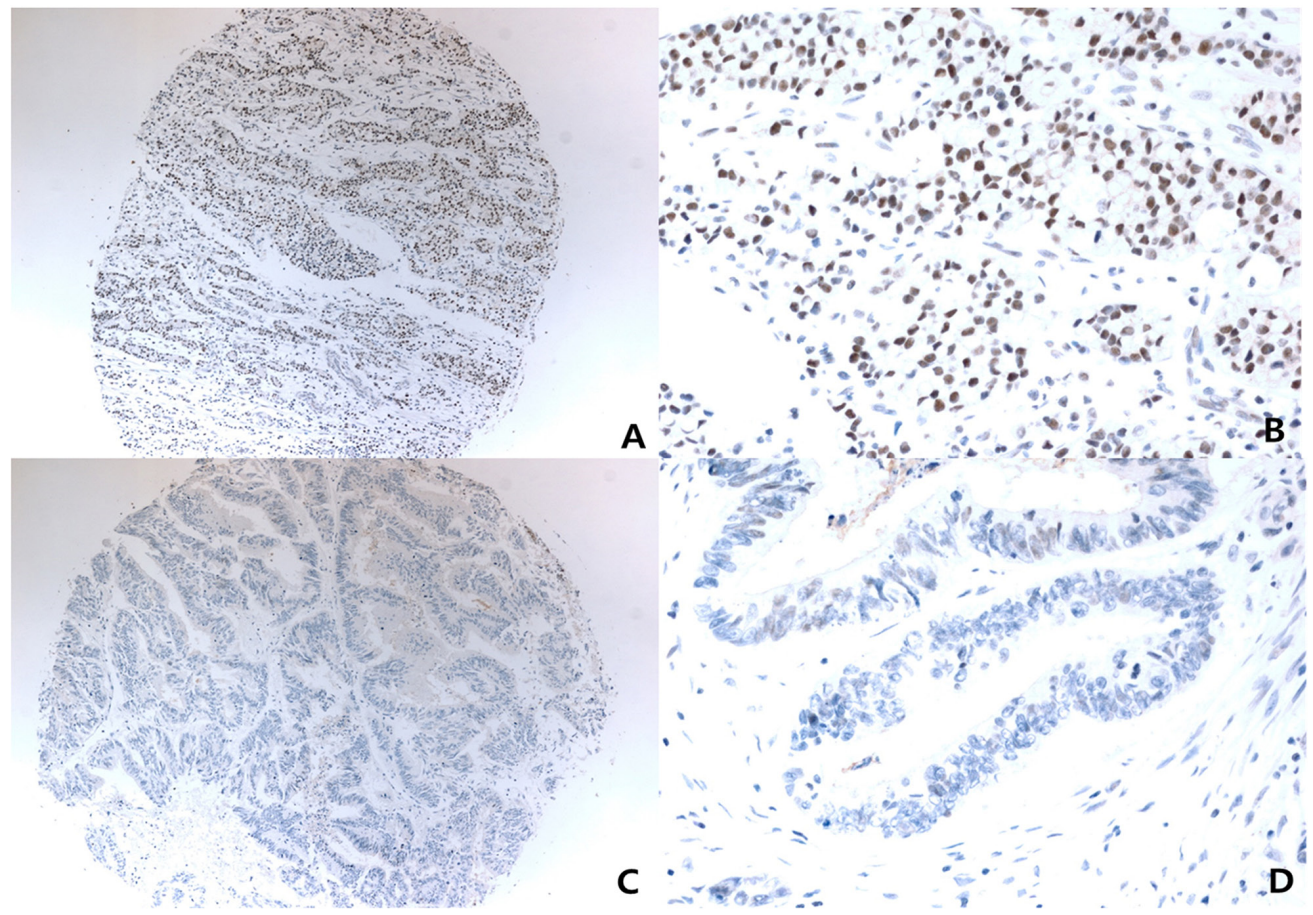
Representative photomicrograph of immunohistochemistry for Anti-MerTK (phospho Y681 + Y749) Low powered view (x40) with strong positive nuclear staining **(A)**, and it's higher powered view (x400) **(B)**, low powered view (x40) with negative nuclear staining **(C)**, and it's higher powered view (x400) **(D)**.

For MerTK interpretation of immunohistochemistry slides, nuclear staining identified in X4 objective lens with moderate-to-strong nuclear reactivity in >50% of tumor volumes were scored as positive. Negative controls without primary antibody were run simultaneously. The slides were assessed by a pathologist blinded to the clinical outcome (Figure 4).

### Western blot analysis

Proteins were extracted from each cell line and equal amount of proteins were separated on 4% to 12% gradient SDS-PAGE. The resolved proteins were transferred onto nitrocellulose membranes, the blots were probed overnight at 4°C with appropriate primary antibodies including p-Axl (Tyr702, #5724, CST, Danvers, MA, USA), Axl (#8661, CST), p-MerTK (Y281+Y749, ab192649, Abcam, Cambridge, MA, USA), MerTK (SC-365499, Santa Cruz, Dallas, TX, USA), p-Erk (#9102, CST), Erk (#4370, CST), p-AKT (#4060, CST), AKT (#9272, CST), Bcl-xL(#2764, CST), Bcl-2(#15071, CST), Bax(#5023, CST), caspase-3(#9662, CST), p-STAT3(Tyr705, #9145, CST), STAT3(#9135), PD-L1(#13684, CST) and β-actin (SC-47778, Santa Cruz Primary antibodies were diluted to 1:500 in TBS containing 0.5% Tween 20 (TBS-T) and then the membranes were washed with TBS-T. Secondary antibody was used for appropriate primary antibody host purchased from CST conjugated with HRP (anti-rabbit IgG (#7074) and anti-mouse IgG (#7076). Secondary antibody was diluted to 1:2000 in TBS-T. Antibody binding was detected using an enhanced chemiluminescence system according to the manufacturer's protocol (Perkin Elmer, MA, USA).

### Statistical analysis

Data were analyzed using the GraphPad Prism 5.0. A paired *t*-test was performed where appropriate and results are expressed as the mean ± standard deviation (SD) or standard error (SE). A p-value < 0.05 was considered statistically significant.
